# Pediatric COVID-19 Follow-Up with Lung Ultrasound: A Prospective Cohort Study

**DOI:** 10.3390/diagnostics12092202

**Published:** 2022-09-11

**Authors:** Domenico Paolo La Regina, Daniela Pepino, Raffaella Nenna, Elio Iovine, Enrica Mancino, Gianmarco Andreoli, Anna Maria Zicari, Alberto Spalice, Fabio Midulla, on behalf of the Long COVID Research Group

**Affiliations:** 1 Department of Maternal Infantile and Urological Sciences, Sapienza University of Rome, 00161 Rome, Italy; 2 Department of Diagnostic Medicine and Radiology, Sapienza University of Rome, 00161 Rome, Italy

**Keywords:** SARS-CoV-2 infection, COVID-19, children, lung ultrasound

## Abstract

During the COVID-19 pandemic, lung ultrasound (LUS) was widely used to assess SARS-CoV-2 infection. To date, there are patients with persistence of symptoms after acute infection. Therefore, it may be useful to have an objective tool to follow these patients. The aim of our study was to evaluate the presence of LUS artifacts after SARS-CoV-2 infection in children and to analyze the associations between time elapsed since infection and symptomatology during acute infection. We conducted an observational study, enrolling 607 children infected with SARS-CoV-2 in the previous twelve months. All patients performed a LUS and medical history of demographic and clinical data. We observed irregular pleural lines in 27.5%, B-lines in 16.9%, and subpleural consolidations in 8.6% of the cases. These artifacts were more frequently observed in the lower lobe projections. We have observed that the frequency of artifacts decreases with increasing time since infection. In symptomatic patients during COVID infection, B-lines (*p* = 0.02) were more frequently found. In our sample, some children, even after months of acute infection, have ultrasound artifacts and showed an improvement with the passage of time from the acute episode. Our study provides additional evidence about LUS in children with previous COVID-19 as a support to follow these patients in the months following the infection.

## 1. Introduction

During the coronavirus pandemic, children are less likely to get infected, and typically they have a less severe illness with fewer deaths [1,2,3]. The most frequent clinical symptom is fever, followed by fatigue, cough, rhinorrhea, sore throat, headache, vomiting, and abdominal pain [4,5]. Although asymptomatic cases in the pediatric population vary in relation to the study analyzed between 15 and 35% [6,7], children with clinical signs of pneumonia represent about 45% of all pediatric cases [8].

In these patients, new evidence from published studies are showing the versatility of lung ultrasound (LUS) from diagnosis to monitoring and follow-up [9,10]. In the last years, this method has been increasingly used and there is growing evidence of its utility in the management of many pediatric lung diseases [11,12,13].

Moreover, during the COVID-19 pandemic, ultrasound has been widely used to assess the severity of severe acute respiratory syndrome coronavirus 2 (SARS-CoV-2) infection in both adults and children.

Lung ultrasound is able to identify alterations affecting air content in the peripheral lung parenchyma. Normally, the air into the lung reflects ultrasound waves completely. Therefore, healthy lung ultrasounds are characterized by horizontal artifacts beyond the pleural line. When the peripheral airspace of the lung is subverted with a reduced tissue/air ratio, ultrasound incident waves could enter acoustic channels and be trapped in acoustic microholes on the pleural plane. This determines the generation of vertical artifacts, resulting in the so-called Sonographic Interstitial Syndrome indicative of a hyperdense preconsolidated state of the lungs [14]. If the tissue/air ratio is further reduced, thickening is generated. On the basis of the above, the main ultrasound findings observable in children with COVID-19 are pleural irregularities, vertical artifacts or areas of white lung, and subpleural consolidations [15,16,17].

It has recently been shown that LUS has a high concordance with the gold standard for diagnosis and assessment of the severity of SARS-CoV-2 pneumonia, computer tomography (CT) of the chest [18,19,20]. Considering that today many studies show that in children, as in adults, there are patients who report the persistence of symptoms after acute infection, defined as Long-COVID [21,22,23], it could be useful to have an objective tool to follow the patients after COVID-19.

Thanks to its wide availability, its safety with the absence of ionizing radiation, its low cost, and its rapidity of execution, LUS can represent an optimal method to follow children with previous COVID-19 infection. This can help to exclude the presence of lung sequelae in patients healed from COVID-19, avoiding them to undergo more invasive tests such as CT.

The primary aim of our study was to assess the presence of artifacts on lung ultrasound in children after months of SARS-CoV-2 infection. The secondary aim was to analyze whether the ultrasound artifacts reduced with the time elapsed from the infection and whether there were differences according to the symptoms during the acute infection.

## 2. Materials and Methods

### 2.1. Study Population

We conducted an observational prospective, single-centre, study from February to November 2021, at the Department of Maternal Science of a tertiary University hospital in Rome. The local Ethics Committee approved the study protocol and informed parental consent was obtained from all patients (RIF.CE 0399/2021).

We enrolled 607 children, 0 to 18 years old, infected with SARS-CoV-2 one to twelve months before enrollment. The infection was documented by a positive nasopharyngeal swab result, performed outside the hospital. Both symptomatic and asymptomatic patients during acute infection were enrolled.

**Figure 1:**
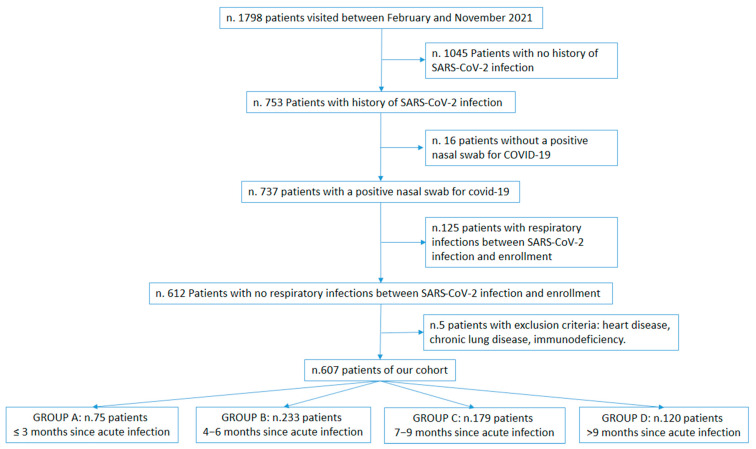
Enrolment flow-chart.

Patients were divided into 4 groups according to the distance from the infection (≤3 months, 4–6 months, 7–9 months, and >9 months) (Figure 1). For each patient, we performed a medical history. With a structured questionnaire, the following detailed demographic and clinical data were collected: age, gender, body mass index, history of respiratory disease, and exposure to smoke. Moreover, we analysed the presence of blood SARS-CoV-2 antibodies. Exclusion criteria were new respiratory infections between SARS-CoV-2 infection and enrollment, congenital heart diseases, immunodeficiency, and respiratory tract malformation.

### 2.2. Lung Ultrasound

LUS was performed by a single experienced radiologist blinded to the patient’s condition using a high frequency (5–12 MHz) linear probe. Ultrasonographic evaluation was performed according to Copetti et al. [24] and the probe was placed vertically, obliquely, and horizontally to the ribs in three chest projections: anterior, lateral, and posterior. For this, we divided the chest into 12 quadrants (two anterior, two lateral, and two posterior quadrants for each hemithorax) and we have analyzed the following ultrasonographic features:

- pleural line morphology;
- identification of B lines and/or “white lung”;
- identification of subpleural consolidations;
- presence of pleural effusion.

In a normally aerated lung, the only detectable structure is the pleura. It appears as a continuous hyperechogenic line that moves back and forth with the breaths (lung sliding). We considered a pleural line with thickening, granularity, and waviness as pathological. Below the pleural line, the healthy lung is filled with air. This does not allow the direct visualization of the normal pulmonary parenchyma, but you can see the “A-lines”. They are horizontal echogenic lines equidistant and parallel to each other and the pleura, representing reverberations of the pleura itself.

B-lines were defined as vertical narrow lines arising from the pleural line. B-lines show a narrow base, extend to the bottom of the screen without fading, and move synchronously with lung sliding.

If the B-lines were less than 3 for quadrant they were not considered pathological. We classified B-lines as multifocal (referring to 3 or more separated B-lines for a quadrant) or confluent.

Finally, the presence of poorly ventilated or solid images near the pleura were identified such as subpleural consolidations. These were divided by size (<0.5 cm, 0.5–1 cm, and >1 cm).

**Figure 2:**
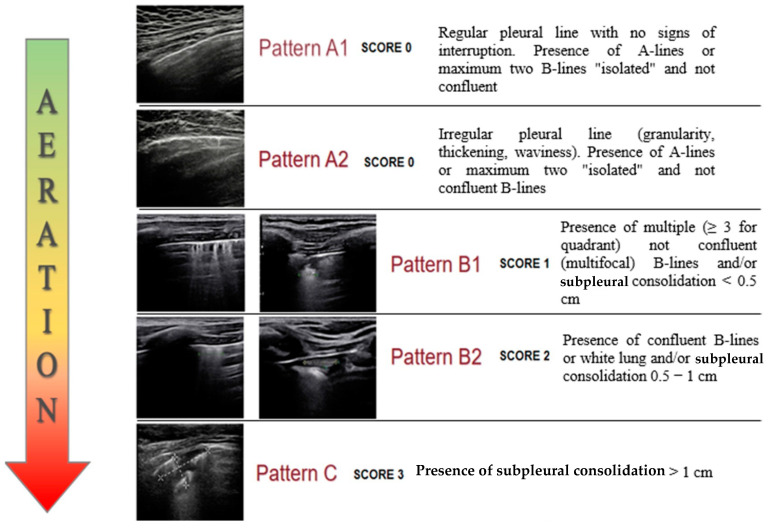
Lung ultrasound patterns with the degree of pulmonary aeration.

Based on these characteristics we identified a modified Soldati-Volpicelli LUS score [10,25] with five patterns of severity, associated with the degree of pulmonary aeration (Figure 2).

### 2.3. Statistical Analysis

The statistical software SPSS (version 27.0; IBM, New York, NY, USA) was used for data analysis. For all the variables studied, we performed a descriptive analysis using percentage values for qualitative variables, and mean and standard deviation values for quantitative variables. For qualitative variables, chi-square tests were used. For quantitative variables without normal distribution, non-parametric Mann-Whitney U Tests were used. Results with *p*-values < 0.05 were considered statistically significant.

## 3. Results

We evaluated 607 consecutive infants (mean age 9.54 ages ± 4.2, 50.9% males) infected with SARS-CoV-2 in the 12 months before enrollment. Table 1 summarized clinical and epidemiological data of patients. In our sample, the most common symptom during the acute phase was fever (47.5%), followed by headache (36.1%), cough (21.9%), ageusia (19.3%), and anosmia (18.9%).

We observed irregular pleural lines in 27.5%, B-lines in 16.9%, and subpleural consolidations in 8.6% of the cases. These artifacts were observed more frequently in the lower lobe projections, particularly patterns B1 and B2 in lower lobes were observed in 80% and 69%, respectively (*p* < 0.001) (Table 2).

A pulmonary quadrant with the presence of white lung was found in one patient while a pleural effusion flap was found in another two patients. Among the ultrasound patterns, the most represented was the A2 pattern, highlighted in 27.5% of cases, while the C pattern was not found in any patient (Table 2).

**Figure 3:**
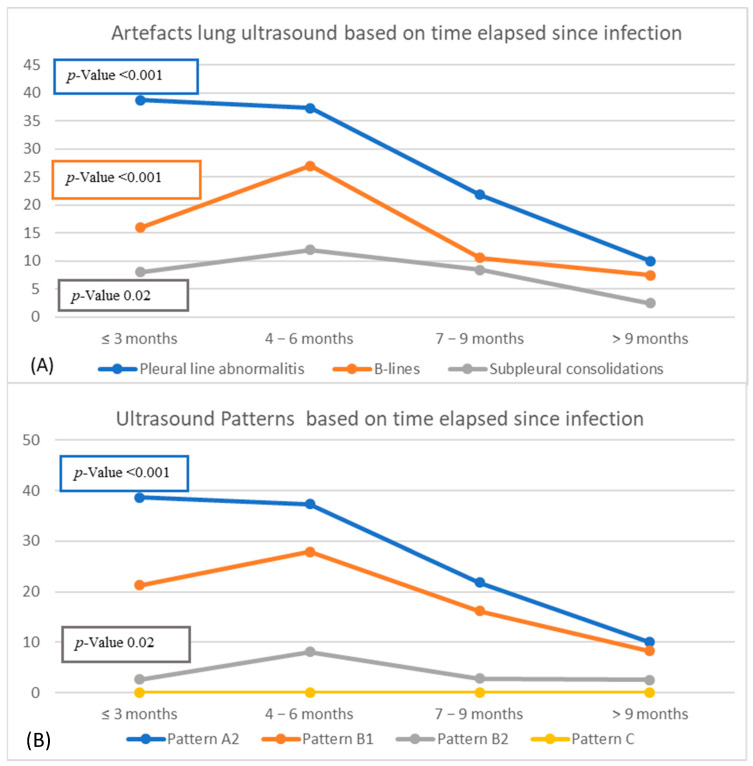
Association between artefacts (**A**) ─ patterns (**B**) lung ultrasound and time elapsed since infection.

We have observed that the frequency of artifacts decreases with increasing time since infection. Patients of “group 4” (infection > 9 months) had fewer ultrasound artifacts than patients of groups with more recent COVID infection (groups 1, 2, and 3) (Figure 3).

In particular, pleural line abnormalities and B-lines were highlighted in 38.7% and 16% of patients within 3 months from the acute infection and in 10% and 7.5% of patients with infection after 9 months (*p* = 0.001). Finally, subpleural consolidations were found in 8% of patients of “group 1” and in 2.5% of patients of “group 4” (*p* = 0.02). Similar results were found while analyzing lung ultrasound patterns.

In symptomatic patients during COVID infection, B-lines (*p* = 0.02) and pattern B1 (*p* = 0.04) were found more often (Table 3).

In our sample, males had more B-lines than females but this result has not found statistical significance. We have found that children with a positive history of asthma had more pleural line abnormalities than patients without asthma (*p* = 0.04). No differences were found in the positive history of exposure to smoke, bronchiolitis, and asthmatic bronchitis.

**Figure 4:**
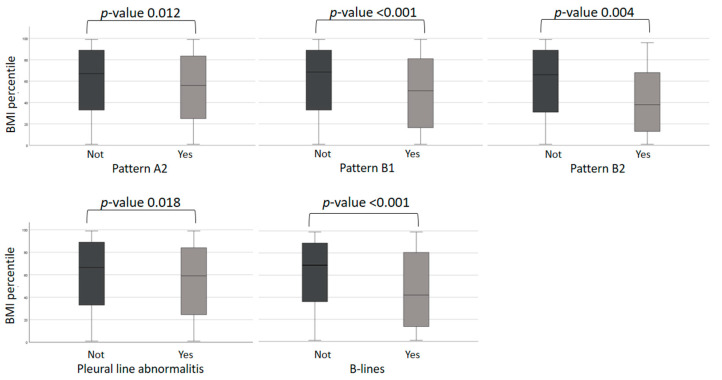
Association between lung ultrasound artifacts and Body Mass Index. Data not statistically significant have not been reported (subpleural consolidation and Pattern C).

Finally, we observed a significant difference in body mass index between patients with and without lung artifacts. In particular, patients with lung artifacts had a lower mean rank of body mass index percentile than patients without artifacts (Figure 4). We did not find other differences based on epidemiological dates.

## 4. Discussion

In our study, we found that a small number of children with previous COVID-19, even after months of acute infection, have ultrasound artifacts compatible with a lung involvement during SARS-CoV-2 infection. We have shown that these artifacts are observed less frequently after time from the acute episode, and they were more frequent in the lower lobes than in the upper lobes. Moreover, we have found more often B-lines and pattern B1 in patients with acute symptoms during SARS-CoV-2 infection. On the other hand, there were no particular differences in the clinical characteristics of the patients except for a difference in body mass index between patients with and without lung artifacts and for pleural line abnormalities in patients with a positive history of asthma.

Since the proposal for more frequent use of LUS in COVID-19 patients [26], there has been increasing evidence in adults that LUS is able to detect artifacts of SARS-CoV-2 infection. However, the main patterns are similar to those that we have described in children.

In our sample, the most frequent LUS findings were pleural lines abnormalities, B-lines, and subpleural consolidation. To date, it is clearly reported which are the main lung ultrasound artifacts during SARS-CoV-2 infection in the pediatric population. Ultrasound artifacts described in our patients are analogous to those described by Musolino et al. [16]. In their study, they enrolled 10 children hospitalized for SARS-CoV-2 infection. In all symptomatic cases, LUS revealed signs of lung involvement during COVID-19 infection. In particular, vertical artifacts, areas of white lung and subpleural consolidations, and pleural irregularities were the main findings in pediatric COVID-19 pneumonia. There were no cases of pleural effusions.

Other pediatric studies showed the presence of pulmonary artifacts at the ultrasound ongoing of COVID-19: Denina et al. and Guitart et al. have reported similar artifacts to ours [27,28].

Sawamura et al. [29] showed that in adults 90 days after the acute infectious episode, there were lung lesions detected at HRTC. Of the 91 patients included, 81% had at least one pulmonary lobe with abnormalities 90 days after discharge. Ground-glass opacities (76%) and parenchymal bands (65%) were the predominant abnormalities. This study confirms our results. Even after months of acute infection, lung lesions are still evident with instrumental investigations.

However, it is important to remember that lung ultrasound findings reflect the common abnormal findings of infection pneumonia. Pleural line irregularities, B lines, and subpleural consolidation are also present in other viral infections.

Moreover, we found that lung artifacts are more frequent in the lower lung lobes. This result is similar to that of other studies [30,31,32]. This may be due to the physiologic regional inhomogeneity of the lung as a result of the influence of gravity [33]. The erect lung is marked by striking regional non-uniformity in perfusion and ventilation resulting in blood flow and ventilation predominating in the lower lobes [34].

The prevalence of lung lesions in the lower regions is not a new concept, but it can also be observed in other viral infections [35].

The secondary aim of our study concerned analyzing possible associations between lung artifacts with time elapsed since the acute infection and the presence of clinical symptoms during acute infection.

We found that the frequency of artifacts decreases with increasing time since infection. The presence of typical viral lung artifacts and the improvement of the ultrasound picture at a distance of time from the infection support the hypothesis that the highlighted artifacts are a direct consequence of a lung involvement during viral infection, still present several months following the acute infection. To avoid mistakes, we have discarded patients with new airway infections in the time elapsed between acute SARS-CoV-2 infection and enrollment.

Our results go in line with the results of Denina et al. [36]. In their study, a clinical-instrumental follow-up was carried out on 25 pediatric patients four months after the acute infection, showing the presence of ultrasound artifacts in the course of follow-up and observing an improvement in ultrasound patterns over time. Although to date there is a lack of studies about the follow-up of SARS-CoV-2 infection in children, taken together, these findings highlight the importance of LUS for pediatric COVID-19 follow-up avoiding the use of ionizing radiation and reducing costs.

In our cohort, we observed B-lines and pattern B1 more often in patients with symptoms during SARS-CoV-2 infection. We also found lung artifacts in asymptomatic patients. This data has already been described in the literature. Ng et al. [37] described CT patterns in 25 asymptomatic young patients who had laboratory-confirmed COVID-19. In their study, chest CT showed abnormalities in six patients (24%), four (16%) had ground glass opacification, one (4%) had a small peripheral subpleural nodule and one (4%) had a dense solitary granuloma. Ng’s results are similar to ours. Whereas the coronavirus is a respiratory virus, it seems plausible that, even in the absence of clear respiratory symptomatology, there is an involvement with the pulmonary parenchyma evidenced by the ultrasound.

Further interesting data emerged from our study. In our cohort, males had more B-lines than females. Even if this result is not statistically significant, it is in concord with that reported in the literature about sex differences in COVID-19 infection [38]. In fact, biological sex differences may manifest themselves in susceptibility to infection, early pathogenesis, innate viral control, adaptive immune responses, or the balance of inflammation and tissue repair in the resolution of infection [38].

Moreover, we have found that children with a positive history of asthma had more pleural line abnormalities than patients without asthma. In the literature, it is reported that patients with asthma had predominant A-lines plus lung sliding [39].

In our opinion, pleural line abnormalities could reflect the exaggerated accumulation of air in the lungs in children with asthma who have an efficient inspiratory drive that is not followed by an efficient expiratory phase due to partially occluded bronchi.

Finally, we observed significant differences in body mass index between patients with and without lung artifacts. In particular, patients with lung artifacts had a lower mean rank of body mass index percentile than patients without artifacts. This result is in contrast with other studies published in the literature, that show an association between BMI and both COVID-19 severity and mortality. Obesity was associated with a significantly increased risk of critical COVID-19 and in-hospital mortality [40]. Our difference may be due to the fact that the lean child has less acoustic impedance during the performance of lung ultrasound and this can improve the vision of artifacts.

Our study has some limits. First of all, only one blinded sonographer without a double-blind control performed LUS examinations. Then, in our study only one lung ultrasound was performed for each child, therefore we were not able to evaluate the evolution of ultrasound patterns in each individual patient. Finally, in our study, we did not have a control group of healthy children.

In conclusion, we can speculate that LUS is a valid tool to investigate pediatric populations with previous SARS-CoV-2 infection. This is possible because SARS-CoV-2 lung lesions are peripheral [41], allowing lung lesions to be identified by ultrasound.

Based on our experience, we consider lung ultrasound to be a safe and harmless method. Some studies have shown ultrasound damage in gas-containing tissues (lungs and intestine), but these experiments were performed on animal models and with acoustic intensity significantly higher than the range commonly used in the diagnostic clinic.

In short, the literature indicates that the bio-effects of ultrasound do not reach clinical levels relevant, and their existence is proven only in in vitro studies and animal models.

For this, to date based on current data in the literature, we believe lung ultrasound is safe and repeatable, especially in pediatrics. This reduces the use of radiation that has been shown to be harmful to the maturing lung. Due to its simplicity of execution, ease of learning, and proven usefulness, we believe that every pediatrician should perform it for the management of the acute phase and the follow-up of patients with infectious lung diseases.

In this context, our study provides additional evidence about LUS in children with previous COVID-19 as a support to follow these patients in the months following the infection. In fact, in our sample, some children, even after months of acute infection, have ultrasound artifacts compatible with a previous SARS-CoV-2 infection and showed an improvement with the passage of time from the acute episode.

If our data were confirmed we should consider revisiting the definition of Long COVID, currently based only on the symptomatological aspect, integrating it with the lung ultrasound findings. Further studies are necessary to support our findings because of the lack of data in the literature regarding LUS in the follow-up of children with previous SARS-CoV-2 infection.

Finally, we recommend performing a lung ultrasound in the follow-up of the pediatric patient with a previous SARS-CoV-2 infection.

## Figures and Tables

**Table 1:** Clinical and demographic characteristics of patients. Variables are expressed as frequencies (percentages) and means (±SD).

Patients with Previous SARS-CoV-2 Infection	n = 607
Age at enrollment, years (SD*SD*)	9.54 (±4.2)
<6 years, n (%)	172 (28.3%)
7–12 years, n (%)	291 (47.9%)
>13 years, n (%)	143 (23.6%)
Sex, male, n (%)	309 (50.9%)
Body Mass Index, percentile (SD)	64.5° (±31.14)
Ab SARS-CoV-2, title (SD)	106 (±98.47)
Time elapsed since acute infection, months (SD)	6.26 (± 2.86)
<3 months, n (%)	75 (12.4%)
3–6 months, n (%)	233 (38.4%)
6–9 months, n (%)	179 (29.5%)
>9 months, n (%)	120 (19.8%)
Exposure to smoke, n (%)	164 (27.0%)
Medical history for:	
Bronchiolitis, n (%)	90 (14.8%)
Asthmatic bronchitis, n (%)	109 (18.0%)
Asthma, n (%)	24 (4.0%)
Atopic dermatitis, n (%)	63 (10.4%)
Allergy to inhalants, n (%)	106 (17.5%)
Symptoms during acute infection, n (%)	478 (78.7%)
Fever, n (%)	289 (47.6%)
Cough, n (%)	133 (21.9%)
Respiratory distress, n (%)	43 (7.1%)
Ageusia, n (%)	117 (19.3%)
Anosmia, n (%)	115 (18.9%)
Vomiting, n (%)	39 (6.4%)
Diarrhea, n (%)	86 (14.2%)
Headache, n (%)	219 (36.1%)

**Table 2:** Rates of patterns and artifacts lung ultrasound at baseline.

Lung Ultrasound Artifacts	n = 607
Pleural line	
Regular Irregular Irregular in only 1 quadrant for hemithorax - Irregular in >1 quadrant for hemithorax	440 (72.5%)
	167 (27.5%)
	57 (9.4%)
	110 (18.1%)
B-Lines	
Absence of B-Lines Presence of B-lines - B-lines in only 1 quadrant for hemithorax - B-lines in >1 quadrant for hemithorax	504 (83.0%)
	103 (17.0%)
	81 (13.3%)
	22 (3.6%)
Subpleural consolidations	
Absence Presence - <1 cm - >1 cm	555 (91.4%)
	52 (8.6%)
	52 (8.6%)
	0 (0%)
White Lung	1 (0.2%)
Pleural effusion	2 (0.3%)
Patterns	
Pattern A2 Pattern B1 - Lower lobes - Upper lobes - Both lobes	167 (27.5%)
	120 (19.8%)
	96 (80.0%)
	12 (10.0%)
	13 (10.0%)
Pattern B2 - Lower lobes - Upper lobes - Both lobes	29 (4.8%)
	20 (69.0%)
	9 (31.0%)
	0 (0%)
Pattern C	0 (0%)

**Table 3:** Association between patterns/artifacts lung ultrasound and symptoms during acute infection.

	Symptoms during Acute Infection		
	Yes (n. 483)	Not (n. 122)	*p*-Value
Pleural line abnormalities	136 (28.1%)	31 (25.4%)	n.s.
B-lines	91 (18.8%)	12 (9.8%)	0.02
Subpleural consolidations	40 (8.3%)	12 (9.8%)	n.s.
White Lung	1 (2.1%)	0 (0%)	n.s.
Pleural effusion	2 (4.2%)	0 (0%)	n.s.
Pattern A2	137 (28.4%)	30 (24.6%)	n.s.
Pattern B1	104 (21.5%)	16 (13.1%)	0.04
Pattern B2	20 (4.1%)	9 (7.4%)	n.s.
Pattern C	0 (0%)	0 (0%)	n.v.

## Data Availability

Data available on request due to restrictions. The data presented in this study are available on request from the corresponding author. The data are not publicly available due to privacy.
